# Induced Higher-order aberrations after Laser In Situ Keratomileusis (LASIK) Performed with Wavefront-Guided IntraLase Femtosecond Laser in moderate to high Astigmatism

**DOI:** 10.1186/s12886-016-0205-5

**Published:** 2016-03-22

**Authors:** Ferial M. Al-Zeraid, Uchechukwu L. Osuagwu

**Affiliations:** Department of Optometry & Vision Sciences, College of Applied Medical Sciences, King Saud University, Riyadh, P.O Box 10219, Riyadh, 11433 Saudi Arabia; Department of Optometry & Vision Sciences, Faculty of Health, Ophthalmic and Visual Optics Laboratory Group (Chronic Disease & Ageing), Institute of Health and Biomedical Innovation, Q Block, Room 5WS36 60 Musk Avenue Kelvin Grove, Brisbane, QLD 4059 Australia

**Keywords:** Laser-assisted in situ keratomileusis, Wavefront-guided, Myopia, Astigmatism, Intralase, Femtosecond laser, Distance visual acuity, Coma, Spherical aberration, Higher-order aberration

## Abstract

**Background:**

Wavefront-guided Laser-assisted in situ keratomileusis (LASIK) is a widespread and effective surgical treatment for myopia and astigmatic correction but whether it induces higher-order aberrations remains controversial. The study was designed to evaluate the changes in higher-order aberrations after wavefront-guided ablation with IntraLase femtosecond laser in moderate to high astigmatism.

**Methods:**

Twenty-three eyes of 15 patients with moderate to high astigmatism (mean cylinder, −3.22 ± 0.59 dioptres) aged between 19 and 35 years (mean age, 25.6 ± 4.9 years) were included in this prospective study. Subjects with cylinder ≥ 1.5 and ≤2.75 D were classified as moderate astigmatism while high astigmatism was ≥3.00 D. All patients underwent a femtosecond laser–enabled (150-kHz IntraLase iFS; Abbott Medical Optics Inc) wavefront-guided ablation. Uncorrected (UDVA), corrected (CDVA) distance visual acuity in logMAR, keratometry, central corneal thickness (CCT) and higher-order aberrations (HOAs) over a 6 mm pupil, were assessed before and 6 months, postoperatively. The relationship between postoperative change in HOA and preoperative mean spherical equivalent refraction, mean astigmatism, and postoperative CCT were tested.

**Results:**

At the last follow-up, the mean UDVA was increased (*P* < 0.0001) but CDVA remained unchanged (*P* = 0.48) and no eyes lost ≥2 lines of CDVA. Mean spherical equivalent refraction was reduced (*P* < 0.0001) and was within ±0.50 D range in 61 % of eyes. The average corneal curvature was flatter by 4 D and CCT was reduced by 83 μm (*P* < 0.0001, for all), postoperatively. Coma aberrations remained unchanged (*P* = 0.07) while the change in trefoil (*P* = 0.047) postoperatively, was not clinically significant. The 4th order HOAs (spherical aberration and secondary astigmatism) and the HOA root mean square (RMS) increased from −0.18 ± 0.07 μm, 0.04 ± 0.03 μm and 0.47 ± 0.11 μm, preoperatively, to 0.33 ± 0.19 μm (*P* = 0.004), 0.21 ± 0.09 μm (*P* < 0.0001) and 0.77 ± 0.27 μm (*P* < 0.0001), six months postoperatively. The change in spherical aberration after the procedure increased with an increase in the degree of preoperative myopia.

**Conclusions:**

Wavefront-guided IntraLASIK offers a safe and effective option for vision and visual function improvement in astigmatism. Although, reduction of HOA is possible in a few eyes, spherical-like aberrations are increased in majority of the treated eyes.

## Background

Laser-assisted in situ keratomileusis (LASIK) has become a widespread and effective surgical treatment to correct myopia and astigmatism [1–6]. Like other corneal refractive surgeries (such as radial keratotomy, photorefractive keratectomy), it is designed to modify the central corneal curvature, making it flatter to correct myopia and steeper to correct hyperopia [7]. This surgical modification might influence the optical quality of the cornea, creating aberrations that will lead to distorted images [8].

Conventional LASIK involved mainly the creation of a stromal flap with the aid of a mechanical microkeratome. Like most standard laser refractive surgery, it eliminates conventional refractive errors (lower order aberration like myopia, hyperopia and astigmatism) leaving higher-order aberrations uncorrected or inducing some higher order aberrations (HOAs) particularly spherical aberrations [3, 9–11] which are thought to be responsible for the patients’ complaints of poor quality of vision, even with visual acuity of 20/25 or 20/20, postoperatively.

Femtosecond laser and wavefront-guided ablations are two new technologies for flap creation [3, 4, 6, 12–14] designed to improve the patients’ quality of vision. Femtosecond laser is a solid-state laser [6, 15, 16] used for flap creation in LASIK procedures. Compared with the conventional LASIK (mechanical microkeratome technology), femtosecond laser create flaps with good predictability of thickness and has rare incidence of flap-related complications [3, 4, 11]. In the wavefront-guided ablation technique, the source of the input data is the objective data from an aberrometer [7] in contrast to the subjective refraction data in the standard excimer treatment. The wavefront-guided ablation technique is targeted at correcting optical aberrations of the eye in order to increase retinal image resolution while offering a more accurate refractive correction with fewer optical side effects than with non-wavefront guided femtosecond laser [1, 7, 17].

High astigmatism is one of the most significant obstacles for achieving satisfactory visual function following refractive surgery [18]. It is associated with high amounts of coma aberrations [19] and affects about 62 % of cases seen in optometry practices [20]. Keratoconus has a high incidence and severity in Saudi Arabia, with an early onset. The disease progresses very rapidly to its severe form at a young age [21] and astigmatism is the hallmark sign of this disease [22]. Wavefront-guided ablations for intraLase treatment has been shown to be effective and predictable in reducing the astigmatism and higher order aberrations [4, 6, 13, 14, 23] in the eye. Assessing the effects of intraLase treatment for treatment of moderate to high astigmatism and higher-order aberrations in our population is important. The aim of this study was to: a), assess the changes in vision and visual outcomes after wavefront-guided IntraLase for high astigmatism; b), evaluate the higher-order aberrational changes using the SCHWIND CAM (Eye-tech-Solutions, GmbH & Co. Kleinostheim, Germany); and c), evaluate the relationship between any observed aberrational changes and the changes in other clinical outcomes, postoperatively. The result could provide a better platform than using sphere and cylinder to evaluate the effectivity of this technique and comparison can be made between this technique and other laser techniques.

## Methods

### Study population

Twenty-three eyes of 15 patients [six males (40 %) and nine females (60 %)] mean age of 25.6 ± 4.9 years (ranging from 19 to 35 years) were randomly recruited from patients already scheduled to undergo the surgery technique in the University hospital. The study was conducted between June 2014 and February 2015. The protocol conformed to the tenets of the Declaration of Helsinki 1975 as revised in Fortaleza 2013 and was approved by the Research Ethics Review Board of the College of Applied Medical Sciences, King Saud University. Before participating in this study, the nature of the study was explained and each patient gave a written informed consent.

Pre-treatment mean refraction spherical equivalent obtained with subjective refraction was −4.12 ± 2.55 D (range from −10.00 to +0.75 D). All patients underwent laser treatment using IntraLase FS60 laser (a 60-kHz platform). Patients were included in this study if they: are aged between 18 and 40 years, had astigmatism above 1.50 D, had no current eye disease or injury, are not on any ocular or systemic medication, and agreed to participate in the study, Soft contact lens wearer had to discontinue contact lens wear 2 weeks prior to surgery and patients were required to come for follow-up examinations up to 6 months after surgery. Astigmatism was defined as moderate for eyes with a cylinder ≥ 1.5 and ≤2.75 D, while high astigmatism was ≥3.00 D [24]. The range of cylinders was from 2.5 D to 4.5 D. Patients were excluded in the presence of any of the following conditions: a systemic or ocular disease likely to influence corneal healing; glaucoma; retinal disorders that might reduce visual acuity (such as myopic maculopathy) or complicate LASIK (eg, equatorial degenerations); history of ocular surgery, or history of dry eyes confirmed by an abnormal Schirmer test.

All surgeries were performed at King Saud University Ophthalmology Department by a single surgeon (ALS). LASIK flaps were created using the 150-kHz IntraLase iFS (Abbott Medical Optics Inc. Santa Ana, CA, USA). A 9.0 mm diameter superior hinge, programmed flap thickness of 105 μm, and an inverted side-cut angle of 130° were created. The bed laser pulse energy was 0.75 μJ with bed separation, spot, and line separations of 7 μm. The side-cut spot and line separation were both set at 5 μm with the same bed laser pulse energy. Postoperatively, patients were evaluated at one day, 1 week, 1 month, 3 months and 6 months. Preoperative and six months postoperative data were used in this study. Postoperative medications included 4 times daily dosage of topical moxifloxacin for 4 days and prednisolone acetate 1.0 % (Predforte) for 7 days.

### Data collection

Clinical evaluation of general and ocular health was performed pre-operatively. For all patients, the same optometrists assessed the following visual parameters, at baseline, after the procedure and at last follow-up (range of 6 – 8 months): uncorrected [UDVA(logMAR)], corrected distance visual acuity [CDVA(logMAR)] obtained by the Snellen projected eye chart; cylinder and sphere by subjective refraction with best sphere maximum visual acuity technique; topographical keratometry values (D), higher-order aberrations [only third to fourth order individual aberrations were considered since they are the most important of the HOAs and are present in higher amounts than other HOAs [25]], and higher-order aberrations RMS were once obtained by SCHWIND Ocular analyzer (Eye-tech-Solutions, GmbH & Co. Kleinostheim, Germany); and applanation tonometry. The wavefront aberration data captured when the entrance pupils were at least 6.0 mm were analysed with a 6.0 mm pupil diameter.

### Statistical analysis

All data were entered into a Microsoft Excel 2007 spreadsheet (Microsoft, Inc, Redmond, Washington, USA) and analysed using the Graphpad Instat software (version 3.00-Graph pad Software Inc., San Diego, CA, USA). A *P* value <0.05 (α) was considered statistically significant, and with 23 eyes the study had a power of 80 % as calculated using the G power software 3.1.3 version. Kolmogorov-Smirnov Test was applied to evaluate the normality of data distribution. Results were presented descriptively as mean and standard deviation (SD) in a table and figure where applicable. The refraction vector components were analysed according to Fourier analysis [26, 27]. The sphere (s), cylinder (C), and axis (θ) were represented as the mean refraction spherical equivalent MRSE, 180° to 90° astigmatism *J*_180_, and 45° to 135° astigmatism *J*_45_ components, calculated with the following equations [27]:

$$ \mathrm{MSER}=\mathrm{S}+\mathrm{C}/2 $$

$$ {J}_{180} = - \left(\mathrm{C}/2\right)\  \cos\ \left(2\uptheta \right) $$

$$ {J}_{45} = - \left(\mathrm{C}/2\right)\  \sin\ \left(2\uptheta \right) $$

To assess the changes in tested parameters postoperatively, Student’s *t*-test was used to compare preoperative and postoperative mean values. To determine whether any higher-order aberration changed differently from the others, the change (Δ) in higher-order aberration calculated as difference between postoperative and preoperative mean HOA value were compared using one way ANOVA. The changes were plotted against the preoperative mean values and the regression coefficient obtained equals the slope of the regression line and gives the predictability metrics for the HOA correction. The mean difference between pre and post-operative HOA were also determined and the associations between the changes in HOAs and preoperative MRSE and astigmatism were tested using Pearson correlation coefficient.

## Results

Table 1 shows the descriptive statistics and results of comparative analysis of refractive components (MSER, *J*_180_ and *J*_45_), visual acuities, keratometry readings and CCT values before and six months after surgery. All patients achieved successful correction (postoperative MRSE = −0.30 ± 0.56 D) showing unaided visual acuity equal or better than 0.00 log-MAR. Data from two patients were not included in this analysis data after they failed to attend the six months postoperative visit.

**Table 1:** Summary statistics (mean ± standard deviation) preoperatively (*n* = 23), six months postoperatively and results of comparative analysis

Measured outcome	Sphere	Cylinder	MRSE	*J*_180_	*J*_45_	UDVA	CDVA	K _steep_	K _flat_	K _average_	CCT
Preop	−2.5 ± 2.5	−3.22 ± 0.59	−4.12 ± 2.55	−0.16 ± 0.92	−0.26 ± 1.36	−0.94 ± 0.35	+0.01 ± 0.09	44.80 ± 1.56	42.16 ± 1.49	43.48 ± 1.47	547.00 ± 21.65
Postop	+0.04 ± 0.48	−0.72 ± 0.46	−0.31 ± 0.56	−0.03 ± 0.25	−0.02 ± 0.35	−0.04 ± 0.07	−0.00 ± 0.02	39.97 ± 2.18	39.06 ± 2.01	39.51 ± 2.08	464.20 ± 48.07
Post-Pre	+2.55 ± 2.65	+2.52 ± 0.69	+3.82 ± 2.82	+0.13 ± 0.94	+0.27 ± 1.39	+0.90 ± 0.35	−0.01 ± 0.09	−4.83 ± 2.36	−3.11 ± 1.93	−3.97 ± 2.12	−82.74 ± 41.45
*P value*	*<0.0001*	*<0.0001*	*<0.0001*	*0.51*	*0.35*	*<0.0001*	*0.48*	*<0.0001*	*<0.0001*	*<0.0001*	*<0.0001*

*MRSE* mean refraction spherical equivalent, *J*_*180*_ *and J*_*45*_ Jackson cross cylinder vector components at 180° and 45° respectively, *CDVA* unaided distance visual acuity, *CDVA* corrected distance visual acuity, *K* keratometry, *CCT* central corneal thickness, *Pre* preoperative and postop = postoperative

### Efficacy & safety

**Fig. 1:**
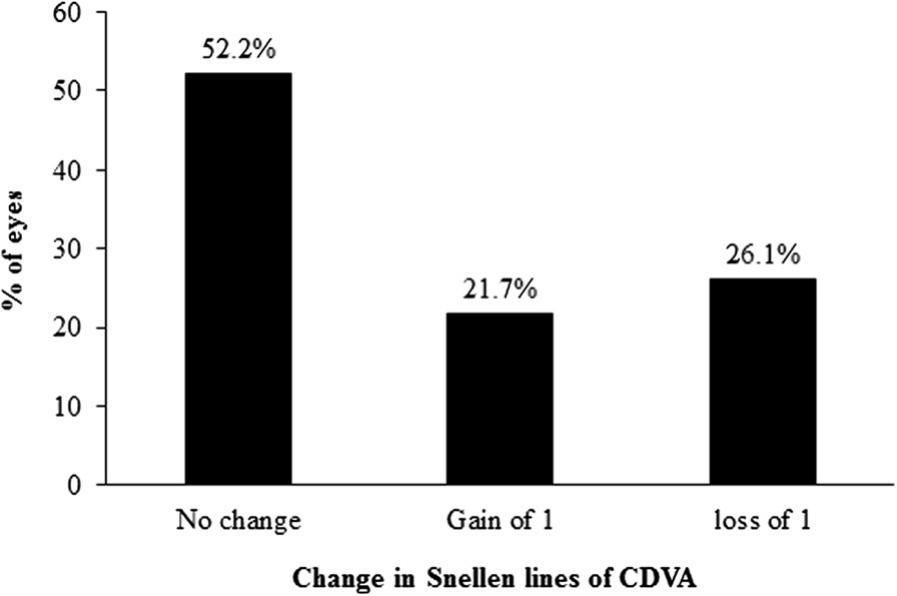
Change in Snellen lines of corrected distance visual acuity (CDVA)

**Fig. 2:**
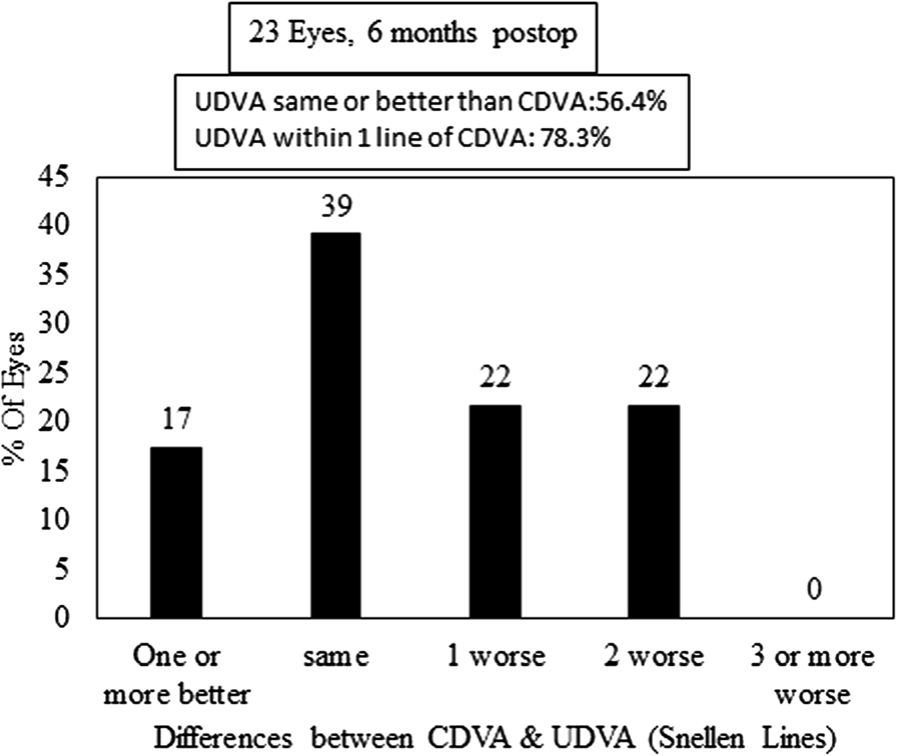
Difference between postoperative uncorrected distance visual acuity and preoperative corrected distance visual acuity

**Fig. 3:**
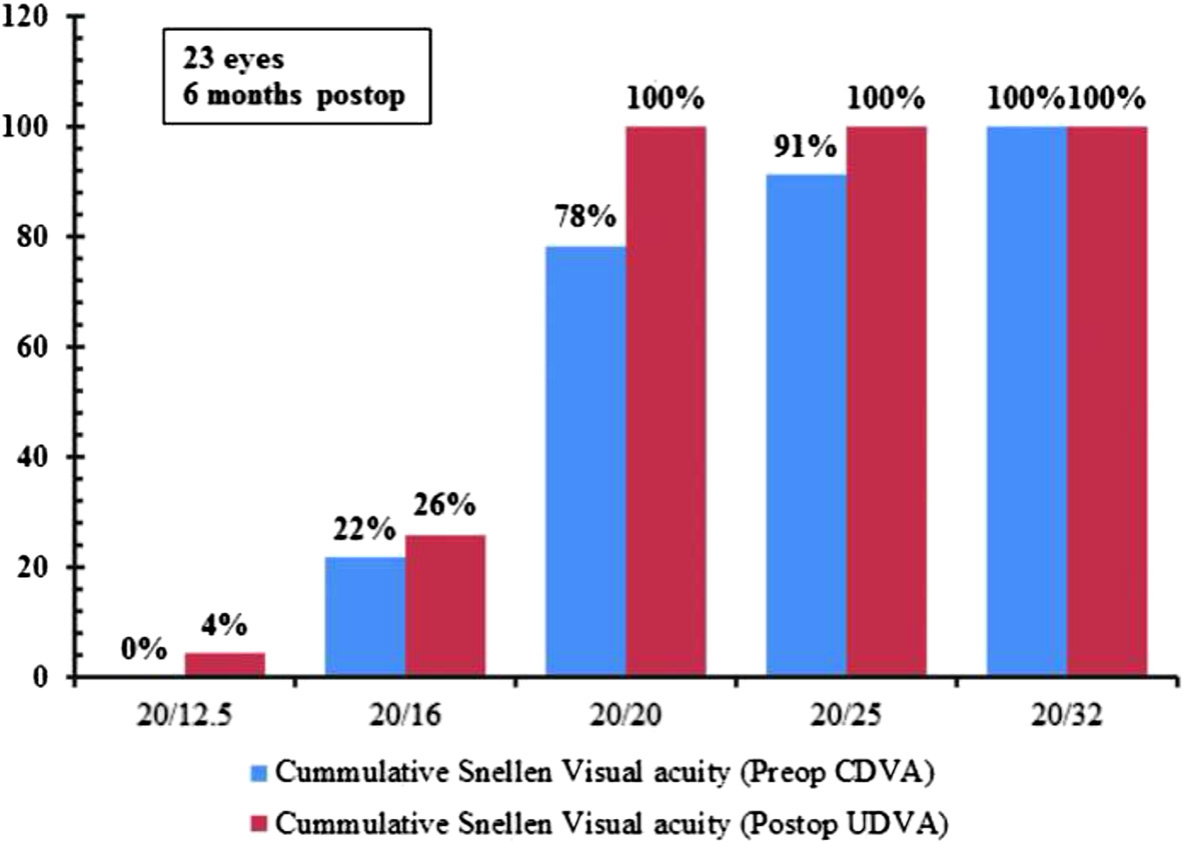
Cumulative Snellen Distance Visual Acuity (Preoperative and Postoperative)

Figure 1 shows the percentage change in CDVA following the procedure. Regarding the method safety, CDVA was unchanged postoperatively (p > 0.05) and remained the same in 12 eyes (52.2 %). Twenty two percent (5/23) of eyes gained at least one line of CDVA and no eye lost ≥2 lines of CDVA. No patient reported any complications at final visit. Preoperative CDVA was not different from postoperative UDVA (Table 1) and none of the eyes experienced supranormal VA of 20/12 or higher. Figure 2 shows the difference between postoperative UDVA and preoperative CDVA. UDVA was the same or better than CDVA in 56 % of eyes and in 78.3 % of eyes, UDVA was within one line of CDVA. Six months postoperatively*,* 69.6 % of eyes had UDVA of 20/20 and 100 % of eyes had 20/32 or better (Fig. 3). As expected, UDVA increased by about 0.90 logMAR (p < 0.0001). The efficacy index was 5.6.

### Predictability

**Fig. 4:**
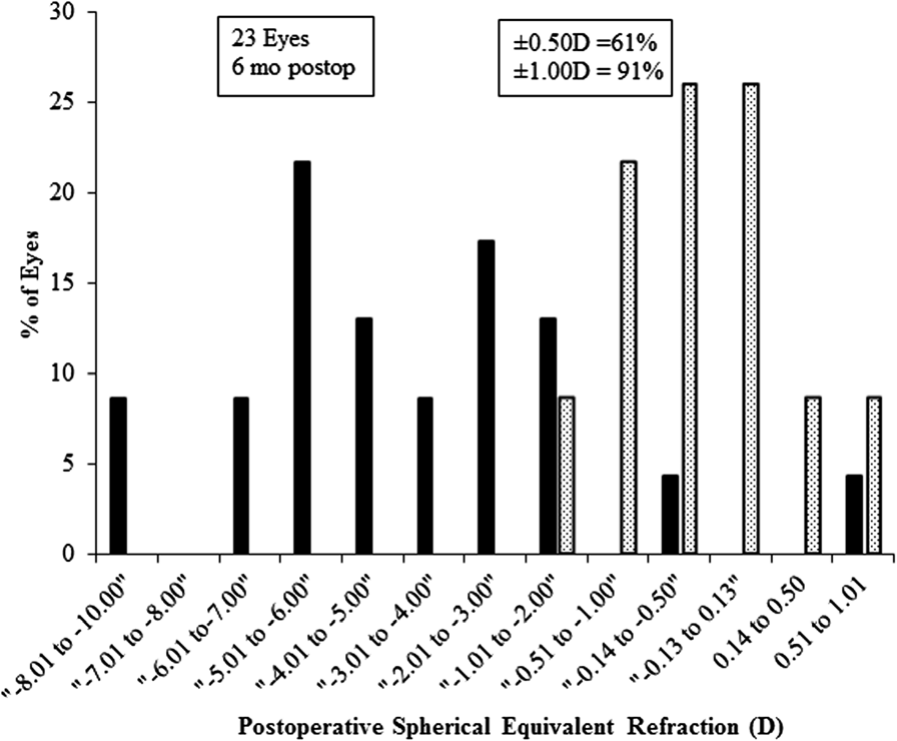
Spherical equivalent refractive accuracy in Dioptres (D)

**Fig. 5:**
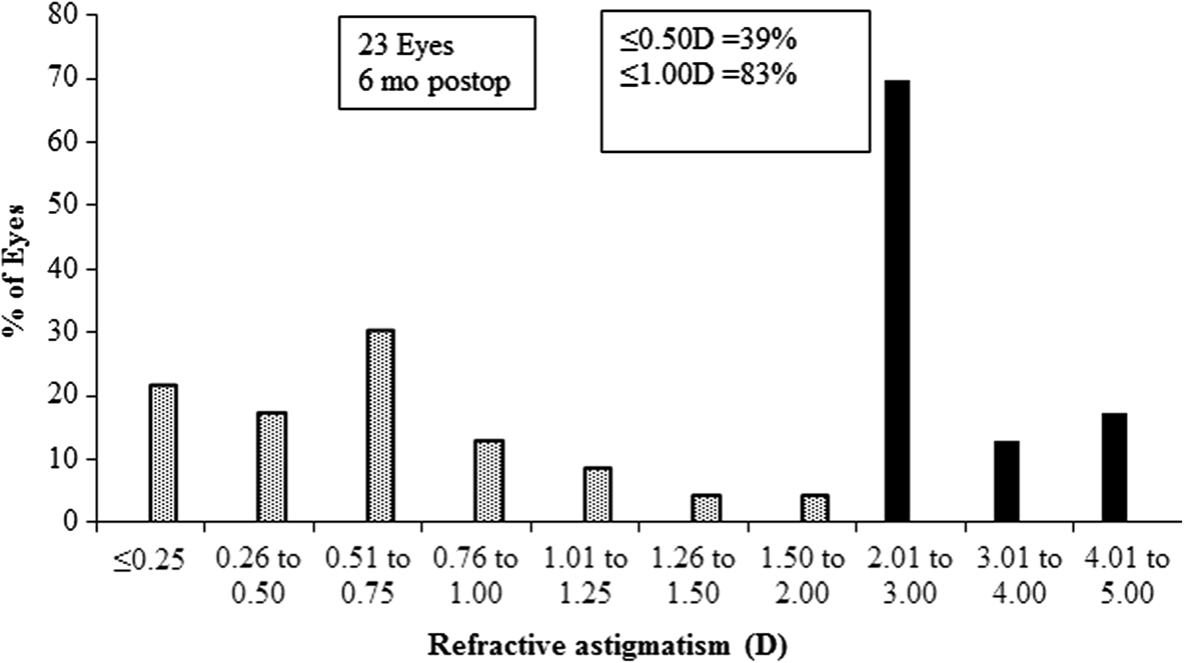
Preoperative and 6 months postoperative refractive astigmatism in Dioptres (D)

The MSER was within ±1.00 in 91 % of eyes, within ±0.50 D in 61 % of eyes and 39 % of eyes achieved absolute emmetropia (+0.50 to −0.25 D), six months postoperatively (Fig. 4). All refractive outcomes were significantly improved (p < 0.0001) except for the *J*_180_ and *J*_45_ which remained unchanged (p > 0.05, for both), postoperatively (Table 1). The mean difference (±SD) between preoperative and postoperative MSER was 3.82 ± 2.82 D (95 % confidence interval of −1.70 to 9.31 D). For sphere refraction, it was 2.55 ± 2.65 D (95 % confidence interval of −2.64 to 7.75D) and for cylinder it was, 2.50 ± 0.69 (95 % confidence interval of 1.14 to 3.86 D). Following the procedure, the postoperative refractive astigmatism was ≤1.00D in 83 % of eyes (Fig. 5).

### Keratometry and central corneal thickness changes

All keratometry readings (K _steep_, K _flat_, K _average_) were significantly decreased (p < 0.0001), six months after the procedure with the corneal curvature becoming flatter by about 4.00 D. Also, the mean CCT was significantly decreased by about 82 μm (p < 0.0001), six months postoperatively (Table 1).

### Changes in higher-order aberrations

**Fig. 6:**
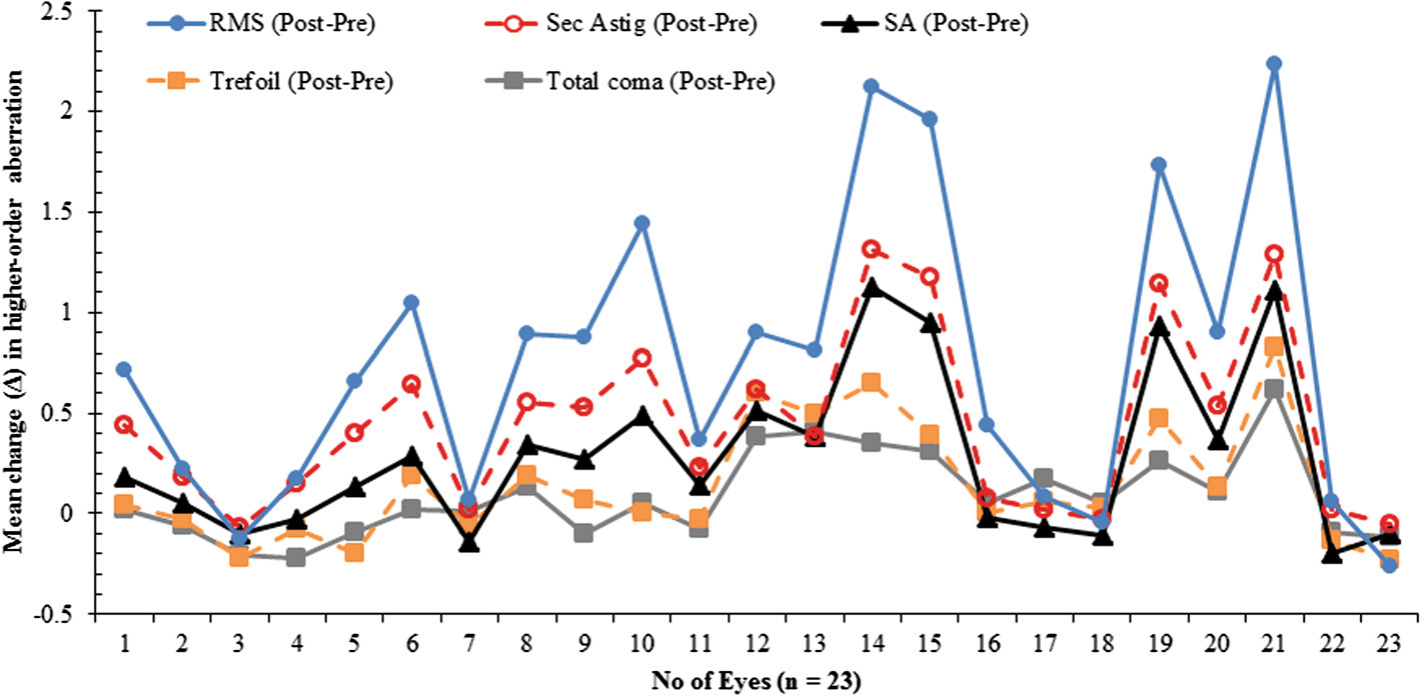
Changes in higher-order aberrations in micrometres (μm), 6 months postoperative

Table 2 shows the preoperative and postoperative mean HOA measurements (over a 6 mm pupil) together with the results of paired *t*-test. There were individual variations in measured higher-order aberration RMS preoperatively (as indicated by the standard deviation of 0.11 μm) which was further widened postoperatively (standard deviation of 0.27 μm). Compared with the preoperative values, the third-order HOAs (coma-like aberrations) were not significantly affected postoperatively although the trefoil aberration just approached a borderline of significance which was considered non-clinically significant. In contrast, the fourth-order aberrations were significantly increased (p < 0.0001), postoperatively. The individual variations in higher-order aberrations six months postoperatively are shown in Fig. 6. The mean higher-order aberration RMS changed significantly (p < 0.0001) postoperatively. The preoperative higher-order aberration RMS ranged from 0.31 μm to 0.76 μm but following the procedure it ranged from 0.40 μm to 1.39 μm. The mean change in HOA was highest for total higher-order RMS (0.3 μm) which was reduced in 13 % of eyes. This was followed by secondary astigmatism and spherical aberration with 0.2 μm mean change for both coefficients. Secondary astigmatism was increased in all eyes postoperatively and spherical aberration was also increased in majority of eyes (69.6 %). Trefoil and coma aberrations were considerably reduced in 39.1 and 34.8 % of eyes. The change in HOAs varied significantly (*p* = 0.0007) but post-hoc analysis using Tukey’s multiple comparison tests (Table 3) found statistically significant differences only in the comparison between changes in higher-order aberration RMS and either coma (p < 0.05) or trefoil (p < 0.05).

**Table 2:** Summary statistics (mean ± standard deviation) preoperatively (*n* = 23), six months postoperatively and results of comparative analysis of higher-order aberrations across a 6 mm pupil

Measured outcome	Coma	Trefoil	Spherical Aberration	Secondary Astigmatism	HOA root- mean-square
Preoperative	0.22 ± 0.11	0.11 ± 0.07	0.18 ± 0.07	0.04 ± 0.03	0.47 ± 0.11
Postoperative	0.31 ± 0.18	0.16 ± 0.10	0.33 ± 0.19	0.21 ± 0.09	0.77 ± 0.27
p-value	0.07	0.047	0.004	<0.0001	<0.0001

**Table 3:** Tukey’s multiple comparison between mean changes (postoperative minus preoperative values) in higher order aberrations (HOAs) postoperative

Change (Δ) in higher-order aberrations	Mean Diff.	95 % CI of diff.	Significant?
Δ Coma vs. Δ Trefoil	+0.03	−0.13 to 0.20	No
Δ Coma vs Δ Spherical Aberration	−0.06	−0.23 to 0.11	No
Δ Coma vs Δ Secondary Astigmatism	−0.08	−0.25 to 0.09	No
Δ coma vs. Δ HOA root-mean-square	−0.22	−0.38 to −0.05	Yes
Δ Trefoil vs Δ Spherical Aberration	−0.09	−0.26 to 0.07	No
Δ Trefoil vs. Δ Secondary Astigmatism	−0.12	−0.28 to 0.05	No
Δ Trefoil vs. Δ HOA root-mean-square	−0.25	−0.42 to −0.08	Yes
Δ Spherical Aberration vs. Δ Secondary Astigmatism	−0.02	−0.19 to 0.14	No
Δ Spherical Aberration vs Δ HOA root-mean-square	−0.16	−0.32 to 0.01	No
Δ Secondary Astigmatism vs. Δ HOA root-mean-square	−0.14	−0.30 to 0.03	No

The Mean difference (Mean diff) and 95 % confidence interval (CI) of mean difference are shown

**Fig. 7:**
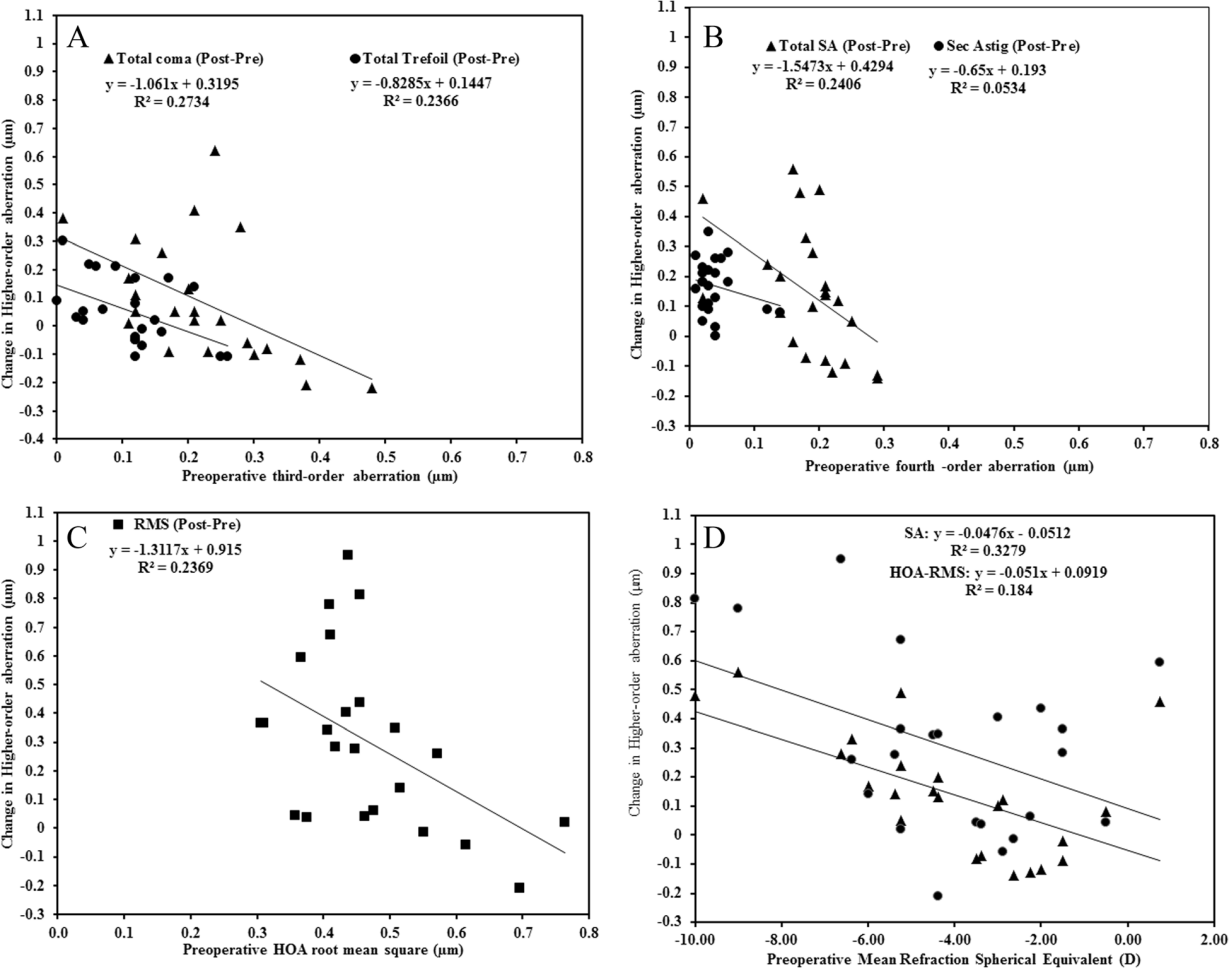
Changes (postoperative minus preoperative) in higher-order aberrations as a function of preoperative aberration values in micrometres (μm; **a** - **c**), and preoperative mean spherical equivalent refraction (**d**)

The preoperative third-order HOAs and the higher-order aberration RMS were moderately related with the change in HOA, following the procedure. For the fourth-order aberrations, a strong relationship was also observed between the preoperative mean spherical aberration and the change in aberration but the relationship between preoperative mean secondary astigmatism and its corresponding change postoperatively, was weak (Fig. 7a-c). Regarding the preoperative MSER (Fig. 7d), it was negatively associated with the change in measured HOAs but this association was significant only with the change in spherical aberration (*r* = − 0.57, *p* = 0.004), and higher-order aberration RMS (*r* = − 0.43, *p* = 0.041) (Table 4). In contrast, the association between preoperative astigmatism and the changes in measured HOAs were not significant (*p* > 0.05, for all).

**Table 4:** Association between changes in higher-order aberrations and preoperative mean spherical equivalent refraction, mean astigmatism, six months following the procedure

Pearson r	Coma (Post-Pre)	Trefoil (Post-Pre)	SA (Post-Pre)	Sec Astig (Post-Pre)	RMS (Post-Pre)
MSER	−0.13 (−0.52 to +0.30)	−0.16 (−0.54 to +0.27)	−0.57 (−0.80 to −0.21)	−0.32 (−0.65 to 0.11)	−0.43 (−0.72 to −0.02)
*p*-value	*0.55*	*0.46*	*0.004*	*0.14*	*0.04*
Astigmatism	−0.18 (−0.55 to 0.25)	−0.36 (−0.67 to 0.06)	−0.08 (−0.48 to 0.34)	−0.38 (−0.69 to 0.03)	−0.31 (−0.64 to 0.12)
*p*-value	*0.41*	*0.09*	*0.72*	*0.07*	*0.15*

Correlation coefficients *r* (95 % confidence intervals) are shown

**Fig. 8:**
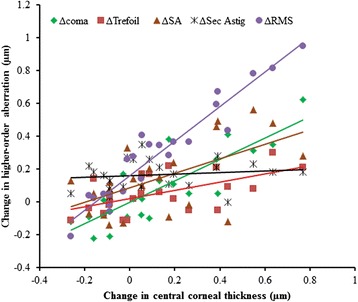
Changes (postoperative minus preoperative) in higher-order aberrations as a function of change in central corneal thickness CCT in micrometres (μm)

The preoperative CCT was not significantly related with any of the measured HOAs (p > 0.05, for all), but the postoperative change in CCT (reduction) was significantly associated with the postoperative increases in trefoil (*r* = − 0.42, *p* = 0.046), spherical aberration (*r* = − 0.59, *p* = 0.003), secondary astigmatism (*r* = − 0.45, *p* = 0.03) and higher-order RMS (*r* = − 0.66, *p* = 0.0007) but not with coma (*r* = − 0.27, *p* = 0.21), Fig. 8.

## Discussion

The 150 kHz iFS Advanced femtosecond laser used in this study is a new generation system. It was shown to be comparable in performance with the Wavelight FS200 system [28]. In this prospective study, the procedure improved reduced all refraction components (MRSE, mean sphere, mean cylinder) except for J_180_ and J_45_ astigmatism vector components which were unaffected. The mean corneal curvature became flatter by about 3.97 D, while the CCT was reduced from 547.00 D to 464.20 D, six months, postoperatively. Refractive astigmatism ranged from −2.50 D to −4.50 D preoperatively, but was considerably reduced by up to −1.75 D, postoperatively. Eighty-three and thirty nine percent of eyes had astigmatism of ≤1.00 D and ≤0.50 D, respectively 6 months following the procedure.

Safety of this procedure (defined as the number and percentage of eyes losing 2 or more lines of CDVA) [1, 29] was excellent as no eyes lost ≥2 lines of CDVA, rather, lines of CDVA were unchanged in one-half of the eyes (12 eyes, 52 %) and in 5 eyes (22 %), a gain of at least one line of CDVA was observed. The safety calculated in this study was slightly better than previous reports six months after LASIK procedure (a loss of two lines of CDVA in 0.6 % of myopic and 0.9 % of astigmatic eyes) in eyes with preoperative sphere and cylinder of up to −11.00 D and −5.00 D, preoperatively [30]. Safety ranges for low to moderate myopia treated with LASIK are between 0 and 7 % and for efficacy, the reported values range from 45 to 79 % for a CDVA of 20/20 [29]. In this study, the safety was calculated to be 5.6 % and the efficacy (defined as the percentage of eyes with an UDVA of 20/20 or better) was 69.6 %. These values are in the upper limits of the reported ranges suggesting that wavefront-guided ablation with IntraLase femtosecond laser is safe and effective for use in the management of eyes with moderate to high astigmatism.

Continuing this, predictability was good in this study, as 61 % of eyes achieved MRSE that was within ±0.50 D while 39 % of eyes achieved absolute emmetropia, postoperatively. A significant hyperopic shift in refraction was observed. The changes observed in this study are comparable with previous reports [1, 30, 31] and are not expected to change significantly after completion of this study. This is because, LASIK eyes were stable from 1 to 3 months after surgery [30, 32].

### Higher-order aberration changes

Patients with moderate to high astigmatism were recruited because of the known presence of above average preoperative higher-order aberrations [33] in these eyes. Although several studies have assessed the wavefront aberrations induced by different LASIK techniques including IntraLase femtosecond laser flap creation, there are no consensus results regarding the changes in individual aberration terms [34]. The difference in the previous reports may be related to the different levels of preoperative aberrations and the pupil analysis diameter used. In agreement with previous reports [1, 32, 35, 36], the preoperative wavefront aberrations in our patients (for a 6 mm pupil) varied widely between patients (SD of 0.11 μm for the higher-order aberration RMS). This inter-individual variation was more pronounced following the procedure (SD of 0.27 μm). The changes in third-order aberrations ranged between −0.34 and 0.51 μm (95 % confidence intervals of mean difference) for coma, and between −0.18 to 0.29 μm for trefoil, but fourth-order aberrations (particularly spherical aberration) were dominant in the eye, postoperatively. Spherical aberration was slightly reduced in few eyes but it increased in majority of eyes by as much as 0.56 μm. Secondary astigmatism was increased in all eyes but the increase was much smaller than previous reports (mean change was 0.4 μm vs 0.2 μm) on wavefront-guided LASIK performed on fewer eyes (*n* = 6) with moderate to high astigmatism [37]. In 87 % of eyes enrolled in this study, higher-order aberration RMS was induced and in few eyes, the increase reached 0.90 μm, postoperatively. These changes in higher-order aberration RMS is consistent with previous reports [1, 8, 11, 32, 38] but it was markedly lower than results from conventional LASIK [3, 8, 11]. Although third and fourth-order aberrations increased only moderately or could be reduced in about one third of the eyes, the increase in spherical-like aberrations was statistically significant. Postoperative spherical aberration increased in eyes with high preoperative higher-order aberration. On the average, all individual higher-order aberrations changed by similar amounts at final visit (Table 3) and the increase in spherical-like aberrations corresponds to the thin central cornea observed in this study, postoperatively [32] (Fig. 8).

Induced HOAs after LASIK procedures have been attributed to various factors including: variations in measurement of HOAs due to fluctuations in accommodation and tear film changes [39]; discrepancy of measurement and treatment position of the eye due to laser misalignment or cyclotorsion [40]; and the ablation rate per excimer pulse since single excimer laser pulse delivered to the cornea which might have different effects at different corneal areas [1]. In the present study, only the spherical-like aberrations were significantly altered, postoperatively and the induced amount was increased as the degree of myopia increased (Fig. 7). This is because, following LASIK, the cornea becomes more prolate as compared to normal corneas [41] and this exposes it to higher amounts of induced spherical aberration [8].

Despite the induced HOAs, the femtosecond laser technique used in this study provided a relatively effective wavefront-guided correction with final outcomes that were not affected by HOA changes. [1, 3, 11, 34, 42]. This technique was shown to yield better postoperative aberration profile than wavefront-optimized LASIK in eyes with higher-order aberration RMS errors >0.3 μm [34]. In this study, the mean preoperative higher-order aberration RMS error was 0.47 μm.

## Conclusions

Wavefront-guided ablation with IntraLase femtosecond laser is a safe and effective option with predictable improvements in visual outcomes in cases with moderate to high astigmatism. Although reduction of higher-order aberration is possible in a few eyes, the technique induced spherical-like aberrations in majority of the treated eyes and increased the higher-order aberration RMS. This increase in higher-order aberrations RMS was linearly related with the degree of preoperative myopia and the postoperative change in central corneal thickness. There is need for further improvement in the predictability of the treatment algorithm used in this procedure.

### Ethics approval and consent to participate

This study was approved by the Research Ethics Review Board of the College of Applied Medical Sciences, King Saud University and all participants gave written informed consent after the study protocol had been explained.

### Availability of data and materials

Data can be shared upon request.

## Abbreviations

logMAR: logarithm of minimum angle resolution
CCT: central corneal thickness
CDVA: corrected distance visual acuity
HOA: higher-order aberration
LASIK: laser in situ Keratomileusis
MSER: Mmean spherical equivalent refraction
RMS: root mean square
SA: spherical aberration
UDVA: uncorrected distance visual acuity
